# Association between sex hormones and bone age in boys aged 9–18 years from China

**DOI:** 10.1111/jcmm.18181

**Published:** 2024-03-20

**Authors:** Wen Shu, Wenquan Niu, Yaqin Zhang, Hui Li

**Affiliations:** ^1^ Department of Growth and Development Capital Institute of Pediatrics Beijing China; ^2^ Children's Hospital Capital Institute of Pediatrics, Chinese Academy of Medical Sciences & Peking Union Medical College Beijing China; ^3^ Center for Evidence‐Based Medicine Capital Institute of Pediatrics Beijing China

**Keywords:** bone age, boy, normal‐weight, overweight and obese, sex hormones

## Abstract

This study aimed to analyse the association between sex hormones and bone age (BA) in boys aged 9–18 years, both individually and interactively, and further to explore whether nutritional status may influence this association. A retrospective analysis was performed among 1382 Chinese boys with physical measurements, sexual characteristics, BA radiographs and sex hormone indicators from February 2015 to February 2022. A total of 470 (34.0%) boys had advanced BA. BA was positively associated with estradiol, luteinizing hormone (LH), follicle‐stimulating hormone (FSH) and testosterone in both advanced and normal BA groups after adjusting for age, genetic height and body mass index. Multiple logistic regression showed that after adjusting for covariates, estradiol (odds ratio [OR] = 1.66, 95% confidence interval [CI]: 1.14–2.12), LH (OR = 1.43, 95% CI: 1.04–1.96), and testosterone (OR = 1.58, 95% CI: 1.17–2.13) were significantly associated with the increased risk of advanced BA in boys, and the association was reinforced when these hormones were interactively explored. Stratified by nutritional status, the interaction between estradiol, LH, and testosterone showed a strong association with advanced BA in boys with normal weight.

## 1 INTRODUCTION

Bone maturity represents the progression from the onset of the ossification center appearance to the morphological changes and enlargement of the epiphysis and metaphysis until the fusion of the epiphysis. It reflects the maturity of physiological development.^1^ In clinical practice, bone maturity is usually justified by bone age (BA),^2^ which is influenced by inherited factors, nutrition, environment conditions, illness^3^ and endocrine factors.^4^ Increasing evidence supports the prominent impact of sex hormones on BA during the onset of puberty.^5^ For example, estradiol and testosterone, produced in increasing amounts during puberty, have been reported to play an important role in regulating bone growth and bone maturation.^6^ However, the association of sex hormones with BA in boys is not fully understood thus far.

With the economic development and improved nutritional status, there is a general trend for early puberty initiation. A systematic review and meta‐analysis published in 2020 revealed a global decrease in age at thelarche by a mean of almost 3 months per decade from 1977 to 2013.^7^ Additionally, several studies have found a trend toward earlier puberty onset in boys.^8^, ^9^ Early puberty onset is inevitably linked with advanced BA, and multiple factors responsible for BA progression may play sex‐specific roles. For example, previous studies showed that overweight and obesity significantly impacted puberty onset and accelerated BA in girls.^10^ However, the effect of overweight and obesity on the timing of puberty and advanced BA in boys remains uncertain. Some studies reported that overweight and obesity may accelerate the rate of BA progression in boys,^11^, ^12^, ^13^ while others failed to support this claim.^14^ Several studies have found that individual hormone levels such as testosterone, dehydroepiandrosterone (DHEA), androstenedione and estradiol independently can accelerate BA in boys.^11^, ^15^, ^16^ Conversely, others have shown that the combined effect of multiple hormones influences skeletal maturation.^17^, ^18^ The inconsistent findings in existing studies might be attributed to the differences in ethnicity, sample size, nutritional status of the populations, investigations timing and the instruments and methods used to measure hormone levels.^19^

To gain comprehensive insights, this study aimed to analyse the association between sex hormones and BA in Chinese boys aged 9–18 years, both individually and interactively, and further to explore the potential effect of nutritional status on this association.

## 2 MATERIALS AND METHODS

### 2.1 Study participants

The study included boys who attended the Growth and Development Clinic at the Children's Hospital of the Capital Institute of Paediatrics during the period from February 2015 to February 2022. Information on physical measurements, sexual development measurements, BA X‐ray and sex hormone indicators were retrospectively collected. Boys with a medical history of a disease that can significantly influence growth and development, with secondary central or peripheral precocious puberty of clear etiology, and with previous use of growth hormone or gonadotropin‐releasing hormone inhibitor drugs were excluded. The final analysis included 1382 boys aged 9–18 years. The research assistants explained to all participants and their legal guardians the extent of medical records usage and obtained their consent. Studies involving human participants were reviewed and approved by the Ethics Committee of the Capital Institute of Paediatrics (Approval number: SHERLL‐2016070).

### 2.2 Sex hormones measurement

Serum estradiol, follicle‐stimulating hormone (FSH), luteinizing hormone (LH), progesterone, prolactin, and testosterone levels were measured by the electrochemiluminescence immunoassay method (Roche Diagnostics GmbH, Mannheim, Germany).

### 2.3 Assessment of outcomes

Two specialized doctors evaluated BA using left‐hand forward radiographs, adhering to the standard method for the hand and wrist skeletal maturity in Chinese children nationality. This method, based on the Tanner‐Whitehouse‐2 (TW2) bone development stages and using healthy Chinese children as a reference sample, is presently the industry standard in the People's Republic of China (JY/T 001–1992).^20^ This approach aligns with the typical growth pattern of Chinese children. If the difference between assessments is below 0.5 years, the average of the two is taken as the final BA. If the difference is 0.5 years or more, a third specialist will evaluate and provide the average of the two assessments with similar results. A difference between BA and chronological age (CA) within the range of −1 to 1 is defined as normal BA, while a difference exceeding 1 is defined as advanced BA.

### 2.4 Collection of covariates

#### 2.4.1 Physical measurements

Height was measured in centimetres, rounded to one decimal place (Seca, model 213, Hammer, Germany). The average of the two measurements was taken with an accuracy of no more than 0.5 cm. Weight was measured using an electronic weight scale (J‐SKY, Jiangsu, China) in kilograms, rounded to one decimal place. Participants were instructed to wear lightweight clothing while measuring height and weight. Body mass index (BMI) (kg/m^2^) was calculated by weight in kilograms divided by the square of height in meters. The z‐scores of BMI and z‐scores of height were calculated based on Chinese children and adolescent growth charts.^21^ BMI was categorized according to the BMI cut‐off points proposed by the World Health Organization. Parental height were obtained by asking or measuring on‐site, and the genetic height of the boy = (father's height + mother's height + 13)/2.

#### 2.4.2 Sexual development measurements

The testicular volumes were measured by a specialist paediatrician using Prader's testicular volume, testicular enlargement equal to or above 4 mL, according to the gold standard in the clinical evaluation of boy pubertal progression.^22^ Pubertal stages are categorized into pre‐puberty, in puberty and completing puberty according to the volumes of the testicles (<4 mL; 4–15 mL; 20–25 mL).

### 2.5 Statistical analysis

Physical measurements and BA are expressed as mean ± standard deviation (SD) and compared between groups using the *t*‐test. Puberty staging was compared using the χ^2^ test. The sex hormone indicators, with skewed distributions, are presented as median (interquartile range, Q1–Q3). The Wilcoxon test was used for comparison between the advanced BA and normal BA groups. Multiple imputations were performed for variables with missing values and 50 complete datasets were obtained for analysis. Complex correlation graphs were used to illustrate the varying levels of sex hormone indicators across different puberty stages. Scatter plots displayed the respective positions of different parameters across all sample sizes, and bar graphs illustrated the distribution of various parameters. Generalized linear models (GLM), adjusting for age, genetic height and BMI, were generated to determine the associations of BA with sex hormone levels in boys, and effect‐size estimates are expressed as odds ratio (OR) and its 95% confidence interval (95% CI). Statistically significant sex hormones were screened by the GLM, and thresholds for sex hormones were determined using restricted cubic splines (RCS) and grouped for logistic regression.

Multiple imputation was conducted using the PROC MI and 50 datasets were generated. A two‐tailed *p* < 0.05 was used to define statistical significance. All data were analysed using the PROC MIANALYZE in SAS V.9.4 M3 (SAS Institute Inc., Cary, North Carolina, USA). The corrplot, PerformanceAnalytics, InteractionR, epiR and rms packages were used to draw complex correlation plots, interaction analysis plots, nomogram and calibration plots in R Studio 2022.12.0 + 353 (RStudio, PBC, Boston, USA).

## 3 RESULTS

### 3.1 Comparison of population characteristics

Total 1382 boys aged 9–18 years were included in this study, with 470 (34.0%) classified in the advanced BA group. Height, weight, BMI, z‐scores of BMI and z‐scores of height were significantly higher in the advanced BA group compared to the normal group (*p* < 0.001). The percentage of pubertal stages was higher in the advanced BA group than in the normal group. Estradiol, LH and testosterone were significantly higher in the advanced BA group than in the normal group. The BA of boys in the advanced BA group was 1.6 years older than that of the normal group (Table 1). Correlation plots between sex hormone indicators at different puberty stages showed that most sex hormone indicators were strongly correlated across all three puberty stages, and thus different puberty stages were analysed as covariates in subsequent analyses (Figure S1).

**TABLE 1:** Demographic characteristics among 9–18‐year‐old boys, China (*N* = 1382).

Characteristics	Normal bone age (*n* = 912)	Advanced bone age (*n* = 470)	*p‐*value
Age, mean (SD), years	11.7 (1.6)	11.5 (1.6)	0.051
Height, mean (SD), cm	145.0 (11.0)	153.0 (11.1)	<0.001
Weight, mean (SD), kg	39.8 (11.4)	49.9 (12.9)	<0.001
BMI, mean (SD), kg/m^2^	18.6 (3.4)	21.1 (3.8)	<0.001
z‐scores of BMI	0.1 (1.1)	0.9 (1.1)	<0.001
z‐scores of height	−0.8 (0.8)	0.6 (1.2)	<0.001
Father height, mean (SD), cm	170.7 (5.5)	171.3 (5.6)	0.070
Mother height, mean (SD), cm	158.6 (5.0)	158.8 (5.2)	0.496
Genetic height, mean (SD), cm	171.1 (4.1)	171.5 (4.3)	0.103
Puberty stage, *N* (%)			<0.001
Pre‐puberty	337 (37.0)	75 (16.0)	
In puberty	489 (53.6)	265 (56.4)	
Completing puberty	86 (9.4)	130 (27.7)	
Estradiol, median (Q1–Q3), pmol/L	18.4 (18.4–42.2)	18.4 (18.4–60.4)	<0.001
LH, median (Q1–Q3), IU/L	1.5 (0.6–2.7)	1.8 (0.6–3.1)	0.007
FSH, median (Q1–Q3), IU/L	3.0 (2.1–4.1)	3.0 (2.1–4.3)	0.461
Prolactin, median (Q1‐Q3), mIU/L	187.8 (140.5–265.2)	192.5 (142.9–270.3)	0.384
Testosterone, median (Q1–Q3), nmol/L	1.2 (0.1–6.7)	2.5 (0.4–11.3)	<0.001
Progesterone, median (Q1–Q3), nmol/L	0.3 (0.2–0.7)	0.3 (0.2–0.7)	0.788
Bone age, mean (SD), years	11.8 (1.7)	13.4 (1.6)	<0.001
Difference bone age‐chronological age, mean (SD), years	0.1 (0.6)	1.9 (0.6)	<0.001

Abbreviations: BMI, body mass index; FSH, follicle‐stimulating hormone; LH, luteinizing hormone; SD, standard deviation; Q1–Q3, interquartile range.

### 3.2 Associations between BA and sex hormones

BA was positively correlated with estradiol, LH, FSH and testosterone in the unadjusted model in boys with advanced BA and the normal BA groups (*p* < 0.001). After adjusting for age, genetic height and BMI, the correlation between BA and estradiol, LH, FSH and testosterone remained positive for boys with advanced BA and those with normal BA (Table 2).

**TABLE 2:** Association of BA groups and sex hormone indicators using generalized linear model among boys aged 9–18, China (*N* = 1382).

Independent variables	Unadjusted model				Adjusted model^a^			
	Normal bone age	Advanced bone age	Normal bone age	Advanced bone age	Normal bone age	Advanced bone age	Normal bone age	Advanced bone age
	Estimate (95% CI)	*p*‐value	Estimate (95% CI)	*p*‐value	Estimate (95% CI)	*p*‐value	Estimate (95% CI)	*p*‐value
Estradiol	0.02 (0.01–0.02)	<0.001	0.02 (0.01–0.02)	<0.001	0.01 (0.01–0.01)	<0.001	0.01 (0.01–0.02)	<0.001
LH	0.59 (0.49–0.68)	<0.001	0.47 (0.37–0.57)	<0.001	0.30 (0.22–0.38)	<0.001	0.31 (0.21–0.41)	<0.001
FSH	0.35 (0.25–0.45)	<0.001	0.30 (0.20–0.40)	<0.001	0.12 (0.05–0.20)	0.001	0.16 (0.07–0.28)	0.001
Prolactin	0 (−0.001–0.002)	0.793	0.001 (−0.001–0.002)	0.500	0 (−0.001–0.001)	0.700	0 (−0.001–0.002)	0.731
Testosterone	0.12 (0.09–0.15)	<0.001	0.10 (0.08–0.12)	<0.001	0.07 (0.05–0.09)	<0.001	0.07 (0.04–0.09)	<0.001
Progesterone	−0.09 (−0.43–0.25)	0.587	0.08 (−0.22–0.38)	0.590	0.03 (−0.17–0.22)	0.792	0.06 (−0.18–0.30)	0.615

*Note*: Estimate, unstandardized estimate.

Abbreviations: 95% CI, 95% confidence interval; FSH, follicle‐stimulating hormone; LH, luteinizing hormone.

^a^ Adjusting for age, genetic height and BMI.

To further explore the relationship between advanced BA and sex hormone indicators, we selected sex hormone indicators that were statistically significant in the GLM and applied the RCS function to determine and group the thresholds for each estradiol, LH, FSH and testosterone (Figure S2). After adjusting for age, genetic height, and BMI, ORs for the estradiol >18.35, LH >1.62, and testosterone >1.31 groups was 1.66 (95% CI: 1.14–2.12), 1.43 (95% CI: 1.04–1.96) and 1.58 (95% CI: 1.17–2.13), respectively, after assigning estradiol ≤18.35, LH ≤1.62. and testosterone ≤1.31 as the reference group (*p* < 0.05) (Table 3).

**TABLE 3:** Association of BA groups and sex hormone indicators using logistic model among boys aged 9–18, China (*N* = 1382).

Significant variables	Unadjusted model		Adjusted model^a^	
	OR (95% CI)	*p*‐value	OR (95% CI)	*p*‐value
Estradiol (pmol/L)				
≤18.35 vs. >18.35	1.48 (1.10–1.97)	0.009	1.66 (1.14–2.12)	0.005
LH (IU/L)				
≤1.62 vs. >1.62	1.24 (0.95–1.62)	0.110	1.43 (1.04–1.96)	0.029
FSH (IU/L)				
≤3.08 vs. >3.08	1.06 (0.81–1.38)	0.677	1.11 (0.82–1.49)	0.506
Testosterone (nmol/L)				
≤1.31 vs. >1.31	1.36 (1.06–1.76)	0.012	1.58 (1.17–2.13)	0.003

^a^ Adjusting for age, genetic height and BMI.

Abbreviations: 95% CI, 95% confidence interval; FSH, follicle‐stimulating hormone; LH, luteinizing hormone; OR, odds ratio.

### 3.3 Interaction of sex hormones on advanced BA

**FIGURE 1:**
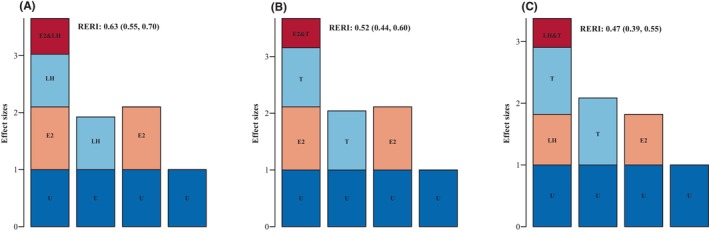
Plot of the two‐by‐two interaction of the four hormones. (A) Estradiol‐LH, (B) Estradiol‐Testosterone, (C) LH‐Testosterone. RERI, relative excess risk of interaction.

ORs for advanced BA among those with estradiol ≤18.35 and LH >1.62, estradiol >18.35 and LH ≤1.62, and estradiol >18.35 and LH >1.62 were 0.92 (95% CI: 0.88–0.96), 1.10 (95% CI: 1.05–1.16) and 1.65 (95% CI: 1.59–1.72), respectively compared to boys with estradiol ≤18.35 and LH ≤1.62 (*p* < 0.05). The relative excess risk due to interactions (RERI) was evaluated to be 0.63 (95% CI: 0.55–0.70), indicating that 0.63 of the relative excess risk was due to interactions (Table 4; Figure 1A). ORs for advanced BA were 1.11 (95% CI: 1.06–1.17) and 1.67 (95% CI: 1.61–1.74) for those with estradiol >18.35 and testosterone ≤1.31 and estradiol >18.35 and testosterone >1.31, respectively, compared to boys with estradiol ≤18.35 and testosterone ≤1.31 (*p* < 0.05). The RERI was estimated at 0.52 (95% CI: 0.44–0.60), indicating that 0.52 of the relative excess risk was due to interactions (Table 4; Figure 1B). Compared to boys with LH ≤1.62 and testosterone ≤1.31, ORs for advanced BA were 1.09 (95% CI: 1.02–1.15), 0.82 (95% CI: 0.77–0.87) and 1.37 (95% CI: 1.33–1.42) for those with LH ≤1.62 and testosterone >1.31, LH >1.62 and testosterone ≤1.31 and LH >1.62 and testosterone >1.31, respectively (all *p* < 0.05). The RERI was estimated to be 0.47 (95% CI: 0.39–0.55), indicating that 0.47 of the relative excess risk was due to interaction (Table 4; Figure 1C).

**TABLE 4:** The two‐by‐two interaction between sex hormones indicators on advanced BA among boys aged 9–18, China (*N* = 1382).

Interaction	OR (95% CI)	*p‐*value
Estradiol‐LH		
Estradiol ≤18.35 and LH ≤1.62	1 [Reference]	
Estradiol ≤18.35 and LH >1.62	0.92 (0.88, 0.96)	<0.001
Estradiol >18.35 and LH ≤1.62	1.10 (1.05, 1.16)	<0.001
Estradiol >18.35 and LH >1.62	1.65 (1.59, 1.72)	<0.001
Estradiol‐Testosterone		
Estradiol ≤18.35 and Testosterone ≤1.31	1 [Reference]	
Estradiol ≤18.35 and Testosterone >1.31	1.04 (1.00, 1.09)	0.076
Estradiol >18.35 and Testosterone ≤1.31	1.11 (1.06, 1.17)	<0.001
Estradiol >18.35 and Testosterone >1.31	1.67 (1.61, 1.74)	<0.001
LH‐Testosterone		
LH ≤1.62and Testosterone ≤1.31	1 [Reference]	
LH ≤1.62 and Testosterone >1.31	1.09 (1.02, 1.15)	0.012
LH >1.62 and Testosterone ≤1.31	0.82 (0.77, 0.87)	<0.001
LH >1.62 and Testosterone >1.31	1.37 (1.33, 1.42)	<0.001

Abbreviations: 95% CI, 95% confidence interval; LH, luteinizing hormone; OR, odds ratio.

### 3.4 BMI‐dependent interaction of sex hormones on advanced BA

Among normal‐weight boys, dose–response relationships were found in estradiol and LH, estradiol and testosterone, and LH and testosterone. Using estradiol ≤18.35 and LH ≤1.62, estradiol ≤18.35 and testosterone ≤1.31 and LH ≤1.62 and testosterone ≤1.31 as the reference group, ORs of advanced BA for estradiol >18.35 and LH >1.62, estradiol >18.35 and testosterone >1.31, and LH >1.62 and testosterone >1.31 were 2.64 (95% CI: 2.50–2.79), 2.70 (95% CI: 2.57–2.84) and 1.89 (95% CI: 1.80–1.98), respectively (all *p* < 0.05). Similarly, the estradiol >18.35 and LH >1.62 and estradiol >18.35 and testosterone >1.31 with ORs of 0.92 (95% CI: 0.86–0.98) and 0.88 (95% CI: 0.83–0.94) in advanced BA (all *p* < 0.05). (Table 5; Figure S3).

**TABLE 5:** Based on BMI grouping of the interaction between sex hormones on advanced BA among boys aged 9–18, China (*N* = 1382).

Interaction	Normal		Overweight and obese	
	OR (95% CI)	*p‐*value	OR (95% CI)	*p‐*value
Estradiol‐LH				
Estradiol ≤18.35 and LH ≤1.62	1 [Reference]		1 [Reference]	
Estradiol ≤18.35 and LH >1.62	1.11 (1.04, 1.18)	<0.001	0.88 (0.82, 0.94)	<0.001
Estradiol >18.35 and LH ≤1.62	1.60 (1.49, 1.70)	<0.001	0.69 (0.64, 0.75)	<0.001
Estradiol >18.35 and LH >1.62	2.64 (2.50, 2.79)	<0.001	0.92 (0.86, 0.98)	<0.001
Estradiol‐Testosterone				
Estradiol ≤18.35 and Testosterone ≤1.31	1 [Reference]		1 [Reference]	
Estradiol ≤18.35 and Testosterone >1.31	1.29 (1.21, 1.37)	0.001	0.86 (0.80, 0.93)	<0.001
Estradiol >18.35 and Testosterone ≤1.31	1.54 (1.43, 1.65)	<0.001	0.71 (0.65, 0.77)	<0.001
Estradiol >18.35 and Testosterone >1.31	2.70 (2.57, 2.84)	<0.001	0.88 (0.83, 0.94)	<0.001
LH‐Testosterone				
LH ≤1.62 and Testosterone ≤1.31	1 [Reference]		1 [Reference]	
LH ≤1.62 and Testosterone >1.31	1.42 (1.31, 1.53)	<0.001	0.79 (0.72, 0.87)	<0.001
LH >1.62 and Testosterone ≤1.31	0.86 (0.79, 0.93)	<0.001	0.92 (0.83, 1.02)	0.100
LH >1.62 and Testosterone >1.31	1.89 (1.80, 1.98)	<0.001	0.98 (0.92, 1.04)	0.060

Abbreviations: 95% CI, 95% confidence interval; BMI, body mass index; LH, luteinizing hormone; OR, odds ratio.

### 3.5 Risk prediction nomogram model

**FIGURE 2:**
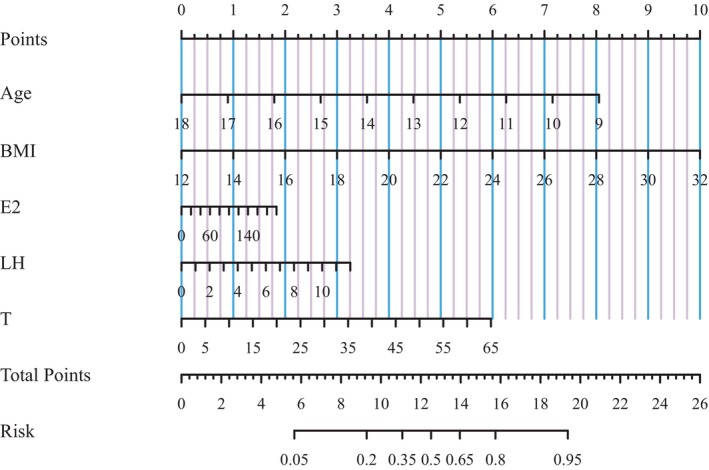
The risk prediction nomogram models.

To enhance the practical application, a risk prediction nomogram model was created for advanced BA in Chinses boys based on the three significant sex hormones identified and some conventional risk factors (Figure 2). The predictive accuracy was good, as reflected by C‐index at 73.2% (*p* < 0.05) and the calibration curve (Figure S4). Taking the risk prediction nomogram model as an example, assuming a boy aged 13.5 years (4 points), with BMI of 22 kg/m^2^ (5 points), estradiol of 140 pmol/L (1.25 points), LH of 2 IU/L (0.5 points), and testosterone of 25 nmol/L (2.25 points), the probability of advanced BA was estimated to be 60%.

## 4 DISCUSSION

This study aimed to analyse the association between sex hormones and BA in boys, both individually and interactively and to explore whether nutritional status can affect this association. It is worth noting that after controlling for confounding factors, we intriguingly identified three sex hormone indicators significantly associated with advanced BA. Importantly, this association was more evident in the presence of pairwise interactions. Furthermore, when study population was stratified according to nutritional status, a pairwise interaction of three hormone indicators was found to be more significantly associated with advanced BA in normal‐weight boys. To our knowledge, this is the first study that has reported an association between the interaction of sex hormone indicators and advanced BA among boys in China.

It is well known that BA is associated with genetic, endocrine and nutritional factors. Our study revealed no statistically significant difference in genetic height between the normal and advanced BA groups. Therefore, genetic factor was only analysed as covariates in this study. Previous studies have highlighted the significant role of endocrine system, particularly certain sex hormone indicators, in influencing BA development in boys. For example, the primary gonadal androgen in boys is testosterone, which significantly influences bone growth and maintenance.^23^ Testosterone is the main substrate for estrogen synthesis, a process that is irreversible. The metabolic actions of testosterone partly result from its conversion to estradiol.^24^ During puberty in boys, estradiol was found to increase simultaneously with testosterone levels and directly correlate with CA and BA, height and weight. This finding confirms the crucial role of estrogens in bone physiology in boys.^25^, ^26^ In particular, estrogens seem to exert a dose‐dependent effect on growth plates.^27^ Actually, low doses of estradiol stimulate ulnar growth in boys, while higher doses inhibit this process of growth.^28^ Yet, most of study populations were originated from Europe and the United States, mainly focusing on Wiedemann–Steiner syndrome^29^ and Madelung deformity,^30^ and often involved small sample sizes and over a long period of time. Taking these considerations into account, this study has examined six sex hormone indicators, BA, anthropometric and sociodemographic information in a relatively large homozygous population. We found that three sex hormones, estradiol, LH and testosterone, were significantly associated with the risk of advanced BA in boys, partially consistent with the results of previous studies. It is of interest to find that LH was also associated with the risk of advanced BA in boys. This association may be attributed to gonadal priming, a process involving the onset of sex steroid production from the gonads in response to pulsatile production of GnRH from the hypothalamus. This, in turn, stimulates the production of LH, which can further stimulate the Leydig cells to produce testosterone.^31^

Another important finding in this study was the evident interaction between estradiol and LH, estradiol and testosterone and LH and testosterone. Subgroup analyses showed that the interaction was more significant in normal‐weight boys. Due to the complexity of the mechanisms associated with advanced BA in boys, the relationship between overweight and obesity, sex hormone indicators and advanced BA remains unclear. Most studies have examined the relationship between specific sex hormone levels and BA in boys or the relationship between the obesity levels and BA progression. In fact, nutrition, sex hormone levels and BA are inextricably linked.^5^ The association of individual sex hormone indicators with advanced BA in boys is modest, and the interaction among sex hormones may be a critical factor in the advanced BA of boys with varying nutritional statuses. To address the limitations of existing studies, we attempted to screen six sex hormones and then select estradiol and LH, estradiol and testosterone, as well as LH and testosterone for interaction exploration. We further stratified them based on nutritional status to conduct a more in‐depth interaction analysis. Our findings suggest that the interaction of the three sex hormones was strongly associated with advanced BA in boys. Notably, this interaction was more significantly associated with advanced BA in normal‐weight boys. This may be attributed to the fact that for overweight and obese boys, advanced BA was influenced by both by sex hormone levels and by the effects of overweight and obesity, which, in turn, also affects sex hormone levels. For instance, testosterone levels in obese boys were normal or slightly higher in prepubertal and early puberty, but significantly lower in the later stages of puberty.^32^ Consequently, sex hormone levels in overweight and obese boys may not be a major factor in advanced BA. In contrast, testosterone production in normal‐weight boys increased with testicular volume throughout puberty, and the production of testosterone remained stable once testicular volume reached 15 mL.^33^ Therefore, the relationship with advanced BA is also more stable, indicating that advanced BA in normal‐weight boys may be associated with endocrine changes.

To enhance the application of our findings, a risk prediction nomogram model was developed for the advanced BA in boys. The model identified a significant effect of BMI on advanced BA, providing further insights into the relationship between overweight and obesity on sex hormone levels and advanced BA in boys, illustrating that overweight and obesity have a more substantial influence on the degree of advanced BA in boys compared to the effect of sex hormones on BA. Nevertheless, further validation is required to determine whether the effects of the three sex hormones, either individually or in interactively, are associated with advanced BA in boys of different nutritional statuses.

The strengths of this study include the use of advanced statistical methods to control for clinical confounders and enhance the quality of clinical information. In addition, the sample size of this study was adequate, and the findings may indirectly explain the relationship between nutritional status, sex hormone levels, and advanced BA in boys. However, some limitations should be acknowledged for this study. First, this was a cross‐sectional study that cannot observe the normal physiologic manifestations associated with accelerated BA during puberty. Although previous study has shown a non‐uniform progression of BA,^34^ the findings were inconsistent. A Polish study revealed that BA began to accelerate after the onset of sexual development, peaking BA at 11.6 years in boys (TW2‐20) compared to 13.8 years in peak height age (PHA), with a peak BA 2 years earlier than in PHA.^35^ Conversely, a Japanese study showed that the age of peak BA in boys (TW2‐RUS: 15.6 years) was 2.7 years later than in PHA (12.9 years).^36^ A mixed longitudinal study conducted in the Netherlands demonstrated that the peak BA in boys was similar to that in PHA.^37^ The results of the existing studies imply that the age of peak BA in boys may be earlier, later or close to the PHA. The inconsistent results may be due to variations in the study populations, the use of different methods to assess BA, and the timing of puberty onset. Although the present study is a cross‐sectional study, the study population was Chinese boys, and the methods used to assess BA were consistent with the Chinese population. The results of the study, combined with other growth and development parameters and laboratory indicators, could provide clinicians with an insight into the status of growth and development of boys at a specific time, which is also of great significance. Second, while the study found that the combined effects of the three sex hormones were associated with advanced BA in boys, it was a single‐centre study and further validation is still needed. Third, the number of overweight and obese boys in our study was relatively low compared to the number of normal‐weight boys, potentially introducing bias to the study. In the future, we will continue to explore the relationship between nutrition, sex hormone levels and BA in boys through longitudinal study, validating the findings in general population surveys.

Taken together, we intriguingly identified a significant association between the three sex hormone indicators and advanced BA in boys. Importantly, this association was more pronounced in pairwise interactions. Moreover, the association between the paired interaction of the three hormones and the advanced BA in normal‐weight boys was significantly stronger. From the perspective of clinical research, this study provides theoretical support for explaining the relationship between nutrition, sex hormone levels and advanced BA in boys.

## 5 CONCLUSIONS

Our findings indicated that estradiol, LH, and testosterone, individually and in interactions, were strongly associated with advanced BA in boys, with the interactions being particularly important. Crucially, we found that the interactions of estradiol and LH, estradiol and testosterone, and LH and testosterone were more strongly associated with advanced BA in normal‐weight boys. Although this finding still requires external validation, it provides an evidence base to explain the relationship between sex hormone levels and advanced BA in boys with different nutritional statuses.

## AUTHOR CONTRIBUTIONS

**Wen Shu:** Conceptualization (equal); data curation (equal); formal analysis (lead); investigation (equal); methodology (equal); project administration (equal); visualization (equal); writing – original draft (lead); writing – review and editing (lead). **Wenquan Niu:** Methodology (equal); validation (equal); visualization (equal). **Yaqin Zhang:** Formal analysis (equal). **Hui Li:** Conceptualization (equal); funding acquisition (lead); supervision (equal); writing – original draft (equal); writing – review and editing (equal).

## FUNDING INFORMATION

This study was supported by the Special Fund of the Paediatric Medical Coordinated Development Center of Beijing Hospitals Authority (XTZD20180403), Public service development and reform pilot project of Beijing Medical Research Institute (BMR2019‐11), and CAMS Innovation Fund for Medical Sciences (CIFMS) (2016‐I2M‐1‐008).

## CONFLICT OF INTEREST STATEMENT

The authors declare that the research was conducted in the absence of any commercial or financial relationships that could be construed as a potential conflict of interest.

## Supporting information


Figure S1.

Figure S2.

Figure S3.

Figure S4.

Data S1.
