# Supplementary figures and images for: Association between sex hormones and bone age in boys aged 9–18 years from China
Source: J Cell Mol Med. 2024 Mar 20;28(7):e18181. doi: 10.1111/jcmm.18181 (PMC10951883; doi:10.1111/jcmm.18181)

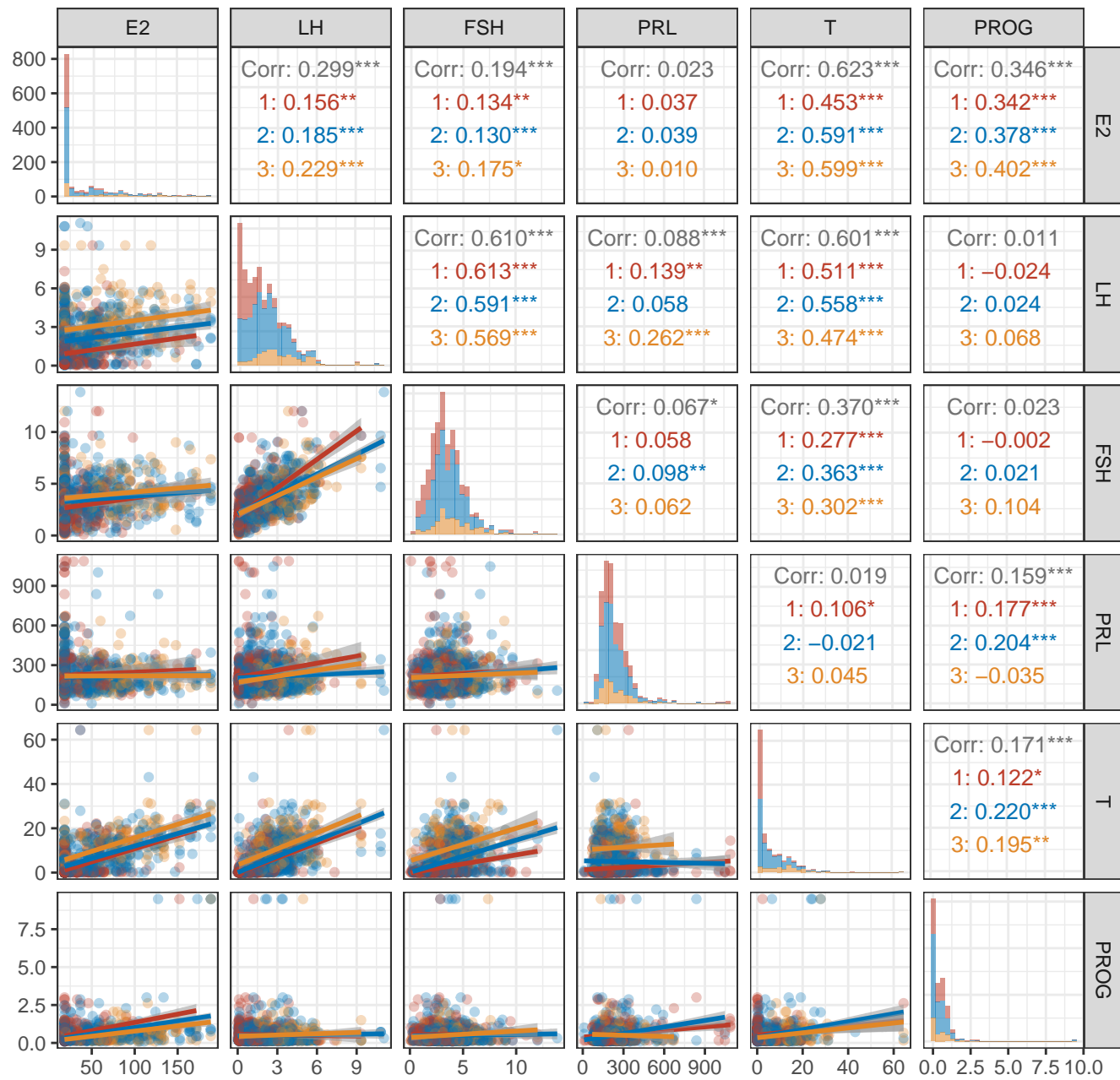

Supplement: Supplementary file 1 — Figure S1. [file JCMM-28-e18181-s005.pdf]

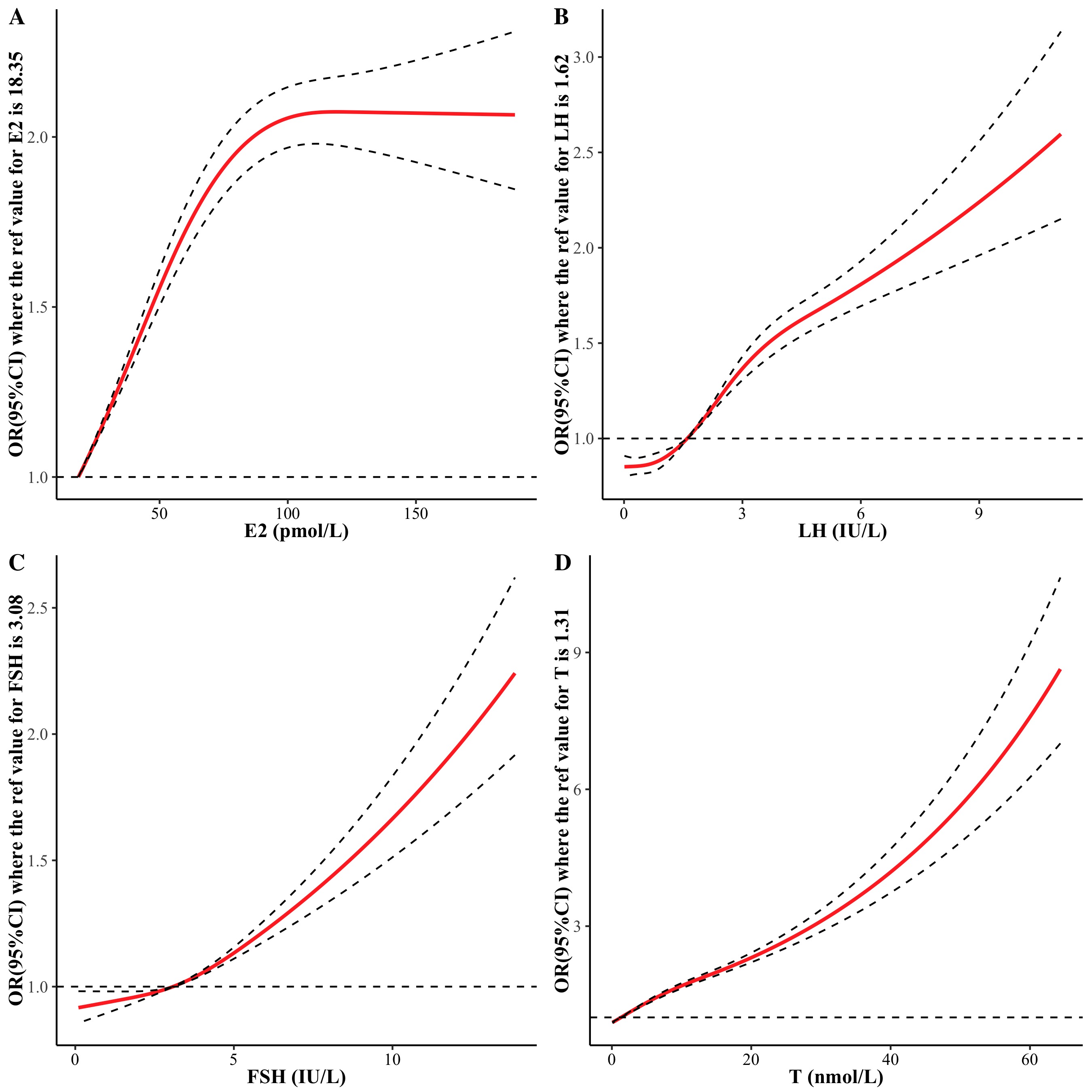

Supplement: Supplementary file 2 — Figure S2. [file JCMM-28-e18181-s002.jpeg]

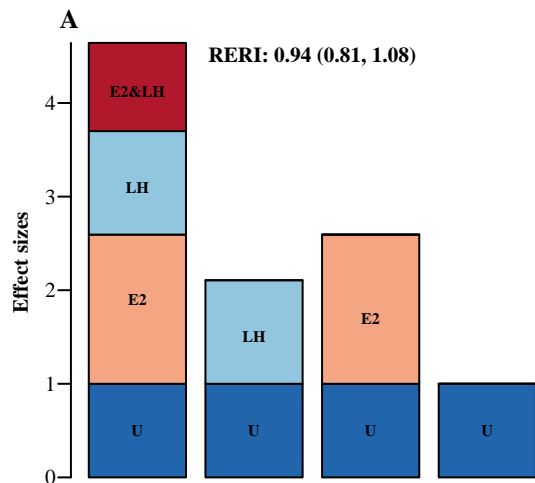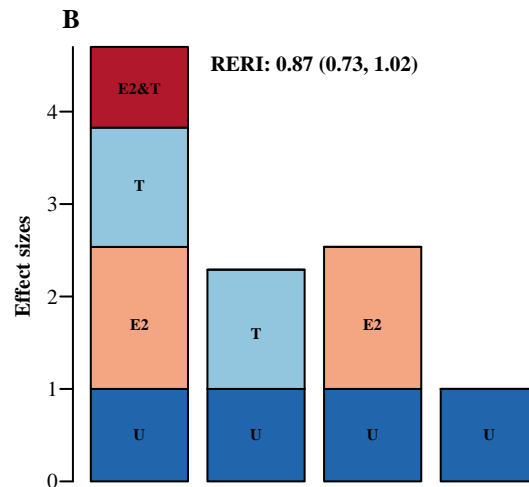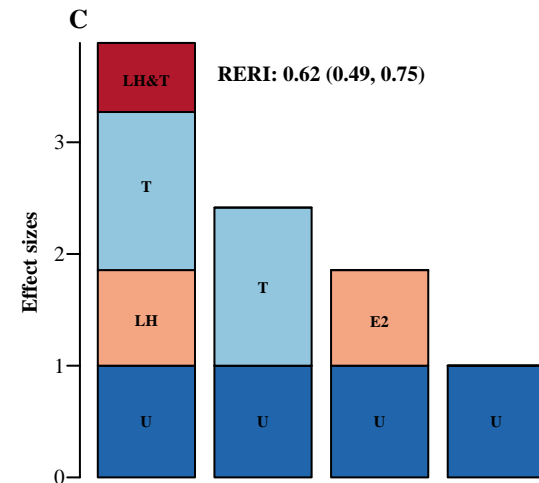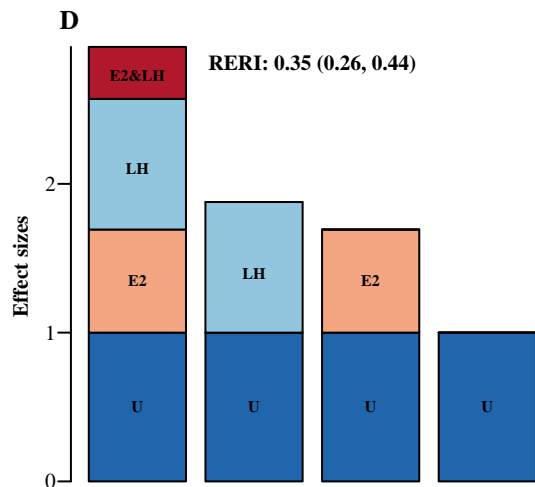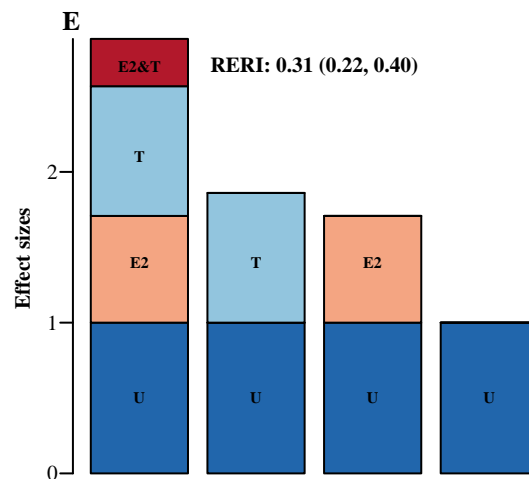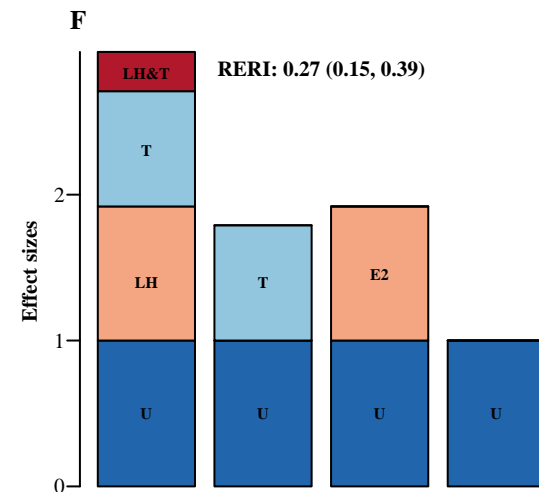

Supplement: Supplementary file 3 — Figure S3. [file JCMM-28-e18181-s003.pdf]

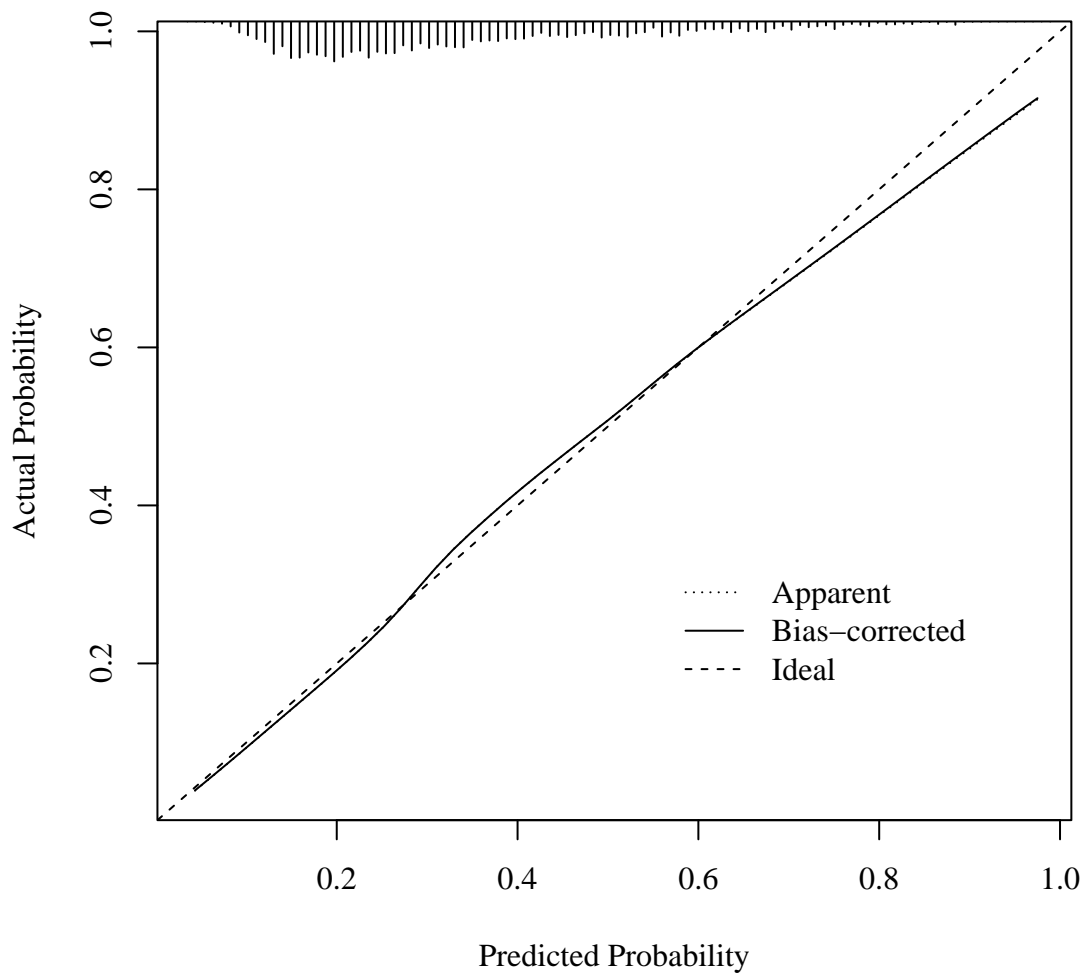

B= 50 repetitions, boot

Mean absolute error=0.01 n=69100

Supplement: Supplementary file 4 — Figure S4. [file JCMM-28-e18181-s004.pdf]
