# Supplementary material for: Association between sex hormones and bone age in boys aged 9–18 years from China
Source: J Cell Mol Med. 2024 Mar 20;28(7):e18181. doi: 10.1111/jcmm.18181 (PMC10951883; doi:10.1111/jcmm.18181)
Supplement: Supplementary file 5 — Data S1. [file JCMM-28-e18181-s001.docx]

Figure S1. Complex correlation graphs of six sex hormones in boys at different pubertal stages. The red line represents pre-puberty, the blue line represents in puberty, and the yellow line represents completing puberty.

Figure S2. Restricted cubic spline plots of (A) Estradiol [E2], (B) LH, (C) FSH and (D) Testosterone [T].

Figure S3. The plot of the two-by-two interaction of the four hormones between normal and overweight and obese boys. (A) Estradiol-LH-normal BMI boy, (B) Estradiol-Testosterone-normal BMI boy, (C) LH-Testosterone-normal BMI boy, (D) Estradiol-LH-overweight and obese boy, (E) Estradiol-Testosterone-overweight and obese boy, (F) LH-Testosterone-overweight and obese boy.

Figure S4. Plot of calibration curve.
